# Serum level of total histone 3, H3K4me3, and H3K27ac after non-emergent cardiac surgery suggests the persistence of smoldering inflammation at 3 months in an adult population

**DOI:** 10.1186/s13148-022-01331-6

**Published:** 2022-09-06

**Authors:** Krzysztof Laudanski, Da Liu, Jihane Hajj, Danyal Ghani, Wilson Y. Szeto

**Affiliations:** 1 Department of Anesthesiology and Critical Care, University of Pennsylvania, JMB 127, 3620 Hamilton Walk, Philadelphia, PA 19146 USA; 2 Department of Neurology, University of Pennsylvania, JMB 127, 3620 Hamilton Walk, Philadelphia, PA 19146 USA; 3 Leonard Davis Institute for Health Economics, University of Pennsylvania, JMB 127, 3620 Hamilton Walk, Philadelphia, PA 19146 USA; 4 Department of Obstetrics and Gynecology, Shengjing Hospital of China Medical University, Shenyang, People’s Republic of China; 5 School of Nursing, Widener University, Philadelphia, PA USA; 6 Department of Cardiac Surgery, University of Pennsylvania, Philadelphia, PA USA; 7 Division of Cardiovascular Surgery, Department of Surgery, University of Pennsylvania, Philadelphia, PA USA

**Keywords:** Histone 3, H3K4me3, H3K427ac, Acetaminophen, Long-term outcome, Smoldering inflammation, Cardiac surgery

## Abstract

**Background:**

Despite clinical relevance of immunological activation due to histone leakage into the serum following cardiac surgery, long-term data describing their longitudinal dynamic are lacking. Therefore, this study examines the serum levels of histone 3 (tH3) and its modifications (H3K4me3 and H3K27ac) alongside immune system activation during the acute and convalescence phases of cardiac surgery.

**Methods:**

Blood samples from fifty-nine individuals were collected before non-emergent cardiac surgery (t_pre-op_) and 24 h (t_24hr_), seven days (t_7d_), and three months (t_3m_) post-procedure to examine serum levels of tH3, H3K4me3, and H3K27ac. Serum heat shock protein-60 (HSP-60) was a surrogate of the cellular damage marker. Serum C-reactive protein (CRP) and interleukin 6 (IL-6) assessed smoldering inflammation. TNFα and IL-6 production by whole blood in response to lipopolysaccharide (LPS) evaluated immunological activation. Electronic medical records provided demographic, peri-operative, and clinical information. Paired longitudinal analyses were employed with data expressed as mean and standard deviation (X ± SD) or median and interquartile range (Me[IQ25; 75%].

**Results:**

Compared to pre-operative levels (tH3_Pre-op_ = 1.6[0.33;2.4]), post-operative serum tH3 significantly (*p* > 0.0001) increased after heart surgery (tH3_24hr_ = 2.2[0.3;28]), remained elevated at 7 days (tH3_7d_ = 2.4[0.37;5.3]), and at 3 months (tH3_3m_ = 2.0[0.31;2.9]). Serum H3K27ac was elevated at 24 h (H3K27ac_24hr_ = 0.66 ± 0.51; *p* = 0.025) and seven days (H3K27ac_7d_ = 0.94 ± 0.95; *p* = 0.032) as compared to baseline hours (H3K27ac_Pre-op_ = 0.55 ± 0.54). Serum H3K4me3 was significantly diminished at three months (H3K4me3_Pre-op_ = 0.94 ± 0.54 vs. H3K27ac_3m_ = 0.59 ± 0.89; *p* = 0.008). tH3 correlated significantly with the duration of anesthesia (*r*^2^ = 0.38). In contrast, HSP-60 normalized seven days after surgery. Peri-operative intake of acetaminophen, but no acetylsalicylic acid (ASA), acid, ketorolac or steroids, resulted in the significant depression of serum H3K4me3 at 24 h (H3K4me3_acetom-_ = 1.26[0.71; 3.21] vs H3K4me3_acetom+_ = 0.54[0.07;1.01]; W[50] = 2.26; *p* = 0.021). CRP, but not IL-6, remained elevated at 3 months compared to pre-surgical levels and correlated with tH3_24hrs_ (*r*^2^ = 0.43), tH3_7d_ (*r*^2^ = 0.71; *p* < 0.05), H3K4me3_7d_ (*r*^2^ = 0.53), and H3K27ac_7d_ (*r*^2^ = 0.49). Production of TNFα by whole blood in response to LPS was associated with serum tH3_24hrs_ (*r*^*2*^ = 0.67). Diminished H3K4me3_24hrs,_ H3K27ac_24hrs_, and H3K27ac_3m_, accompanied the emergence of liver failure.

**Conclusions:**

We demonstrated a prolonged elevation in serum histone 3 three months after cardiac surgery. Furthermore, histone 3 modifications had a discrete time evolution indicating differential immune activation.

## Background

Anesthesia, tissue damage, reperfusion, hypoxemia, thermal shock, and exposure to large artificial surfaces trigger a complex inflammatory reaction during cardiac surgery [1–4]. Induction of apoptosis, necrosis, and other forms of cell damage result in the release of danger-associated molecular patterns (DAMP), including heat shock proteins (HSP), high mobility group box-1 (HMGB-1), adenosine triphosphate (ATP), DNA, RNA, and S100 proteins [5–11]. The total amount of released DAMP gauges the destructive degree of the iatrogenic surgical insult [12]. Further, DAMP modulates inflammation by triggering toll-like receptor-mediated (TLR) responses via the TLR4 receptor [5, 6, 13]. This response may be especially detrimental in a patient with a pre-existing over-activation of the immune system, as DAMP-mediated immune-activation may represent a “second hit,” triggering organ failure [6, 8, 9]. Leukocyte hyporesponsiveness often accompanies this second hit to bacterial challenges like lipopolysaccharide (LPS) [1, 14–19]. Post-surgical process of tissue repair and healing involves significant apoptosis, necrosis, and remodeling and may lead to leakage of histone into the systemic circulation [2, 8, 10, 20–22]. Subsequent persistence of elevated serum histones may sustain smoldering inflammation in the aftermath of cardiac surgery [2, 4, 9–11, 15, 23].

Prior research has demonstrated elevated serum histone levels up to three days post-operatively [12, 23]. However, if the histone leakage persists longer, the smoldering inflammation will lead to progressive organ dysfunction, accelerated arteriosclerosis, graft failures, and congestive heart failure [1, 4, 16, 24]. These comorbidities significantly limit the long-term surgery aimed at improving myocardial oxygenation and quality of life. Despite their importance, the persistence of histones in circulation after three days after the initial surgical insult is unclear [23, 24]. The data demonstrated the predictive value of histone change in determining a composite score of complication post-surgery, but the underlying cause was not explored. Smoldering inflammation, abnormal immune system reactivity, diminished histon moderators, or direct toxic effects of histones may be the causes. Duration of post-surgical histone elevation will be a critical factor in determining the progression of these changes.

However, there are no data on baseline levels of histones or their composite landscape in cardiac surgery patients despite several factors in histone milieu [9, 25, 26]. Pre-existing conditions affect the makeup of circulating histones in numerous illnesses [5–8, 11, 20, 25, 27, 28]. Coronary artery disease, one of the most common indications for coronary artery graft bypass (CABG) surgery, has a specific make-up of atherosclerosis-related histones [26]. Most data did not account for peri-surgical management or peri-operative medications. The latter is of particular importance as several medications can affect the epigenetic landscape, which has not been accounted for in prior studies [23, 24, 29–31]. The need to relate pre-existing diseases and patients’ conditions before surgery with subsequent changes in histone levels after cardiac surgery necessitates longitudinal studies [20, 26, 30, 32, 33]. These studies are challenging to conduct. Finally, an analysis of the epigenomic landscape should be conducted holistically, incorporating several histone modifications and their clinical correlates [10, 28, 34]. This type of analysis is mainly seen in cardiac diseases treated pharmacologically, with the paucity of data focusing on surgical interventions in general or cardiac surgery in particular [12, 23, 25, 34].

DAMP-mediated immune activation can be mitigated by direct or indirect defensin mechanisms limiting histones' detrimental effect [11, 35–38]. One type of defensin is C-reactive protein (CRP), which competes at phospholipid binding sites, directly interrupting the formation of toxic histone complexes and their integration with the cellular wall and calcium influx complexes [8, 9, 38]. CRP also indirectly mitigates the effect of circulating histone toxicity by limiting intravascular coagulation, reducing endothelial damage, and scavenging free radicals [39–42]. Consequently, disruption of CRP mechanisms combined with a significantly high level of histones, in general, represents potentially unfavorable conditions for patients [38]. Unfortunately, few data is analyzing both the inflammatory effects of extracellular histones and defensins (CRP) together [11, 23, 38]. Considering that CRP's role in cardiovascular illness is frequently ambiguous, the lack of definite benefit or harm triggered by CRP changes may depend on the concomitant histone dynamics [6, 38, 39, 41–44].

The evolution of peri-operative inflammation impacts the long-term resolution of the inflammation and outcomes of cardiac surgery or any critical care illness [14, 15, 33]. Prior studies demonstrated that trimethylation of histones in position 3 (H3K4me3) is linked to immunomodulation, while acetylation in position 27 is linked to the emergence of immuno-activation (H3K27ac) [20, 45–48]. Both modifications are related to long-term reprogramming of the immune system [15]; however, it is unclear if modifications of circulating histones reflect overall immune system activation or acute peri-operative inflammation since allostatic immunosuppression may occur during recovery from surgical insult [4, 12, 16, 24]. Considering that histones can be released during apoptosis and necrosis, measuring their level in the blood can reveal the potential of the serum to serve as the vehicle for immune system activation [3, 7, 40].

This study investigated knowledge gaps regarding the long-term release of histones after cardiac surgery. We hypothesized that histone 3 (H3) would be released predominantly during the acute period after cardiac surgery, similar to other DAMP (HSP-60) [21, 22]. Considering the immunostimulatory effect of circulating histones, we hypothesized that the level of circulating histones would correlate with immune system activation (serum IL-6, peripheral monocyte MO activation in response to bacterial challenge) [1, 16, 24, 26, 28, 43, 46, 49]. Furthermore, we theorized that patients’ histone modification profiles would change from peri-operative pro-inflammatory to convalescent anti-inflammatory. Finally, we hypothesized that changes in the serum levels of total H3 (tH3) and its modification could be linked to post-operative end-organ failure [5–10, 23].

## Methods

### Patient recruitment

A total of 59 patients were enrolled in this observational study. The University of Pennsylvania Institutional Review Board approved the study (#815,686). All patients scheduled for non-emergent cardiac surgery were approached for consent. Exclusion criteria were < 18 years of age, emergent surgery, lack of consent, and transplant or immunosuppressed patients.

### Clinical data collection

Demographics and clinical data were obtained from electronic health records (EHR), including surgical, anesthesia, and peri-operative records. The Charlson Comorbidity Index (CCI) was calculated to assess chronic disease burden [50]. Acute Physiology And Chronic Health Evaluation II (APACHE II) score was calculated upon admission to the intensive care unit (ICU) as well as 24 and 48 h later [51]. Organ failures (liver failure, central nervous system failure, cardiovascular failure, acute kidney failure) were defined using the Multiorgan Dysfunction Score (MODS) framework [52]. Serum values of N-terminal pro-B-type natriuretic peptide (NT-proBNP) as the measure of congestive heart failure, and troponin, the measure of pre-existing active ischemia of the heart, were collected from EHR based on the laboratory values obtained by the primary team [3, 9, 23, 53, 54]. The incidence of pulmonary embolism (PE), deep venous thrombosis (DVT), and cerebrovascular accident (CVA) were extracted from EHR. Mortality was defined at 28 days and 3 months.

### Study procedure

After consent was secured, patient blood was collected before the onset of the surgery (t_Pre-op_). Subsequent blood procurements took place 24 h (t_24hr_) and seven days (t_7d_) post-surgery, with a final follow-up at three months (t_3m_).

Blood was collected from arterial lines during the hospital stay, central lines' venous system, or manually drawn using the Vacutainer™ system (BD; Franklin Lakes, NJ). Blood was collected in heparinized tubes and stored at 4 °C for up to 2 h. Plasma was obtained after 5 min at a 1200x*g* spin and stored at − 80 °C until needed.

### Serum histone and inflammatory marker measurements

Circulating levels of tH3, H3K27ac, and H3K4me3 were measured using enzyme-linked immunosorbent assay (ELISA) kits (Epigentek; Farmingdale, NY) and read on BioTek Synergy H1 (BioTek Instrument Inc., Winooski, VT) at 450 nm with 570 nm correction. CRP was also measured with an ELISA kit (Sino Biological; Wayne, PA) and read on BioTek Synergy H1 (BioTek Instrument Inc., Winooski, VT) as specified above. TNFα, IL-6, and HSP-60 were measured using a multiplex assay (Thermo Fisher, Philadelphia, PA) and analyzed on 3D FlaxAmp (Thermo Fisher, Philadelphia, PA).

### General immune activation measurements

A total of 0.5 mL of whole blood obtained at all time points was stimulated with lipopolysaccharide (LPS) [50 ng/mL] (Lonza, Wayne, PA) or left unstimulated for 18 h at 37 °C. Plasma was obtained after 5 min at a 1200xg spin, and supernatants were collected. TNFα and IL-6 were measured with ELISA kits (BioLegend; San Diego, CA) and read on BioTek Synergy H1 (BioTek Instrument Inc., Winooski, VT).

### Statistical analysis

Shapiro–Wilk W and K-S tests were used to test the normality of the distribution variables and assess the distribution of these variables. Parametric variables are expressed as mean and standard deviation (X ± SD) and compared using *t*-test for two variables, while ANOVA was used for multiple comparisons. For nonparametric variables, median and interquartile ranges (*M*_*e*_;[IR]) were utilized with the U-Mann–Whitney test employed to compare such variables. *r*^2^ and *ρ* Spearman correlation coefficients were used to assess relationships for parametric and nonparametric variables, respectively. *k*-means clustering was employed where appropriate. Both-sided *p*-values less than 0.05 were considered statistically significant for all tests unless a specific null hypothesis was formulated. Statistical analyses were performed with Statistica 11.0 (StatSoft Inc., Tulsa, OK) or Statistical Package for the Social Sciences v26 (IBM, Amon, NY).

## Results

### Patient characteristics

A total of 59 patients were enrolled in the study. Their demographic, clinical, and peri-operative characteristics are presented in Table 1. The study group is representative of the patient cohort in a major academic center.

**Table 1:** Patient demographics, clinical, and peri-operative characteristics

Patient demographics	(*N* = 59)
Age (X ± SD [years])	65.1 ± 11.16
Age above 60 [(% of total])	
Males (n [% of total])	39 (66.1%)
BMI (X ± SD [kg/m^2^])	2.2 ± 0.89
Race (% of total)	
* Caucasian*	84.7%
* Black*	5.1%
* Asian*	5.1%
* Other*	5.1%
*Anesthesia and surgery data*	
Duration of anesthesia (mean ± SD [min])	370.3 ± 91.72
Duration of surgery (mean ± SD [min])	257.8 ± 76.82
Coronary artery bypass surgery (*n*)	31
Extracorporeal circulation (% of total; duration [X ± SD (min)]	89%; 127.6 ± 58.2
Aortic-cross-clamp (% of total; duration [X ± SD (min)]	86%; 87.9 ± 43.9
Mitral valvuloplasty and replacement (*n*)	10
Aortic valvuloplasty and replacement (*n*)	20
Aortic aneurysm repair (n)	7
Others (*n*)	4
Estimated Blood Loss (X ± SD [mL])	180.7 ± 216.39
*Peri-operative management*	
* Transfusions during surgery*	
Packed Red Blood Cells (mean [CI 95%] [mL])	94.8 (0;1200)
Fresh Frozen Plasma (mean [CI 95%] [mL])	48.2 (0;750)
Cell Server (mean [CI 95%] [mL])	640.8 (0;1350)
Total crystalloid during surgery (X ± SD [mL])	1365.7 ± 544.08
* Clinical Care 24 h post-surgery*	
Packed Red Blood Cells (mean [CI 95%] [mL])	21 (0;600)
Fresh Frozen Plasma (mean [CI 95%] [mL])	0 (0;0)
Corticosteroid Administration (% of total)	10.2%
Ketorolac Administration (% of total)	3.4%
Acetaminophen Administration (% of total)	79.7%
ASA Administration (% of total)	72.9%
*APACHE scores during ICU stay (X ± SD)*	
1 h	18.6 ± 5.96
24 h	10.2 ± 5.72
48 h	9.9 ± 5.57

Comorbidities	(% of total)
CCI (median [95% CI])	4(1.6;2.4)
Acute Coronary Syndrome	15.25%
Chronic heart failure	15.25%
Connective tissue disease (non-active)	5.7%
Cerebrovascular disease	10.2%
Type 2 diabetes	37.3%
AIDS	1.6%
COPD	8.47%
Leukemia/neoplasms	0%
Acquired Immunodeficiency Syndrome	0%
*Outcomes*	
Mortality	5.08%

SD = standard deviation; BMI = body mass index; CI = confidence interval; ASA = ; APACHE = acute physiological assessment and chronic health evaluation; CCI = Charlson Comorbidity Index; AIDS = acquired immunodeficiency syndrome; COPD = chronic obstructive pulmonary disease

Age (over 60 years old) or gender did not differentiate pre-op H3Kme4, or H3K27ac but tH3 level before was higher in older subjects (tH3_over60_ = 1.7 ± 2.22 versus tH3_under60_ = 4.1 ± 5.72; *t* [59] = 2.37; *p* = 0.021). Neither pre-existing comorbidity (coronary artery disease, congestive heart failure, COPD, diabetes), nor cumulative CCI score significantly impacted circulating levels of tH3, H3K4me3, or H3K27ac (data not shown) [50]. There was no correlation between tH3 and its modifications with pre-operative NT-BNP. Only pre-operative H3K4me3 correlated with serum troponin (*r*^2^ = 0.45; *p* = 0.038).

The baseline serum level of tH3 of any studied modification was not differentiated in a statistically significant way if a patient underwent CABG vs non-CABG surgery (data not shown).

### Serum histone levels after cardiac surgery

**Fig. 1:**
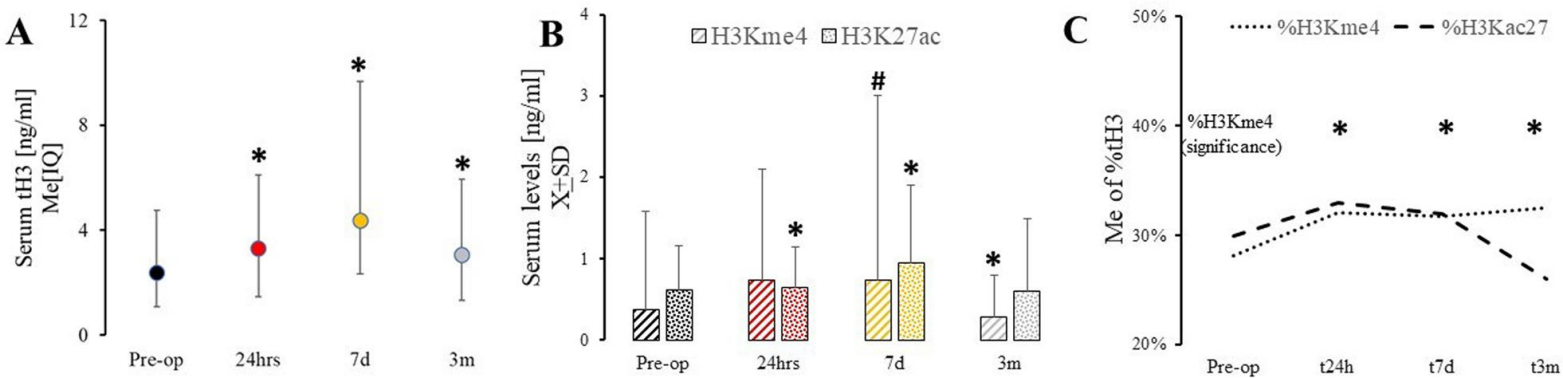
Histone levels pre- and post-cardiac surgery. **A** Serum tH3 levels before surgery (Pre-op), and 24 h (t24h), 7 days (t7d), and 3 months (t3m) after surgery; Histone modifications H3K4me3 (stripes) **B** and H3K27ac (dots) **C** at Pre-op, t24h, t7d, and t3m; and changes in %tH modifications over time before and post cardiac surgery (C). *two-sided *p* < 0.05; #one sided *p* < 0.05

Compared to pre-operative levels (tH3_Pre-op_ = 1.6[0.33;2.4]), post-operative serum tH3 significantly (F[59;3] = 34.4; *p* > 0.0001) increased after heart surgery (tH3_24hr_ = 2.2[0.3;28]), remained elevated at 7 days (tH3_7d_ = 2.4[0.37;5.3]), and 3 months (tH3_3m_ = 2.0[0.31;2.9]) (Fig. 1A). The older subjects had less elevated levels of tH3 at 24 h and 7 days but not 3 months (Appendix Fig. 7).

In comparison with pre-op level, H3Kme4 was borderline increased in serum at 7 days (H3Kme4_Pre-op_ = 0.94 ± 1.217 versus H3Kme4_7d=_1.53 ± 0.94; t[57] = − 1.88; *p* = 0.032 one-sided) to be significantly diminished at 3 months (H3Kme4_Pre-op_ = 0.94 ± 1.217 *vs.* H3Kme_3m_ = 0.52 ± 0.51; t[51] = 2.87; *p* = 0.0061) (Fig. 1B). The proportion of tH3 modified as H3K4me3 (%H3K4me3) demonstrated a borderline increase from pre-surgical levels to 24 h (*p* = 0.037; one-sided) and 7 days post-surgery (*p* = 0.0078), and three months (*p* = 0.028) (Fig. 1C).

Serum H3K27ac was elevated at 24 h (H3K27ac_24hr_ = 0.66 ± 0.51; *t*[57] = 2.25; *p* = 0.028) and seven days (H3K27ac_7d_ = 0.94 ± 0.95; *t*[58] = 3.012; *p* = 0.0039) as compared to baseline hours (H3K27ac_Pre-op_ = 0.55 ± 0.54) (Fig. 1B). %H3K27ac was highly variable in the longitudinal analysis at any time point and nonsignificant (Fig. 1C).

When examining correlations between patient demographics and clinical characteristics with serum levels of tH3 and its modifications, age correlated with tH3_24hr_ (*r*^2^ = − 0.61; *p* < 0.05) and tH3_7d_ (*r*^2^ = − 0.55; *p* < 0.05), but not H3K4me3 or H3K27ac at any time points.

**Fig. 2:**
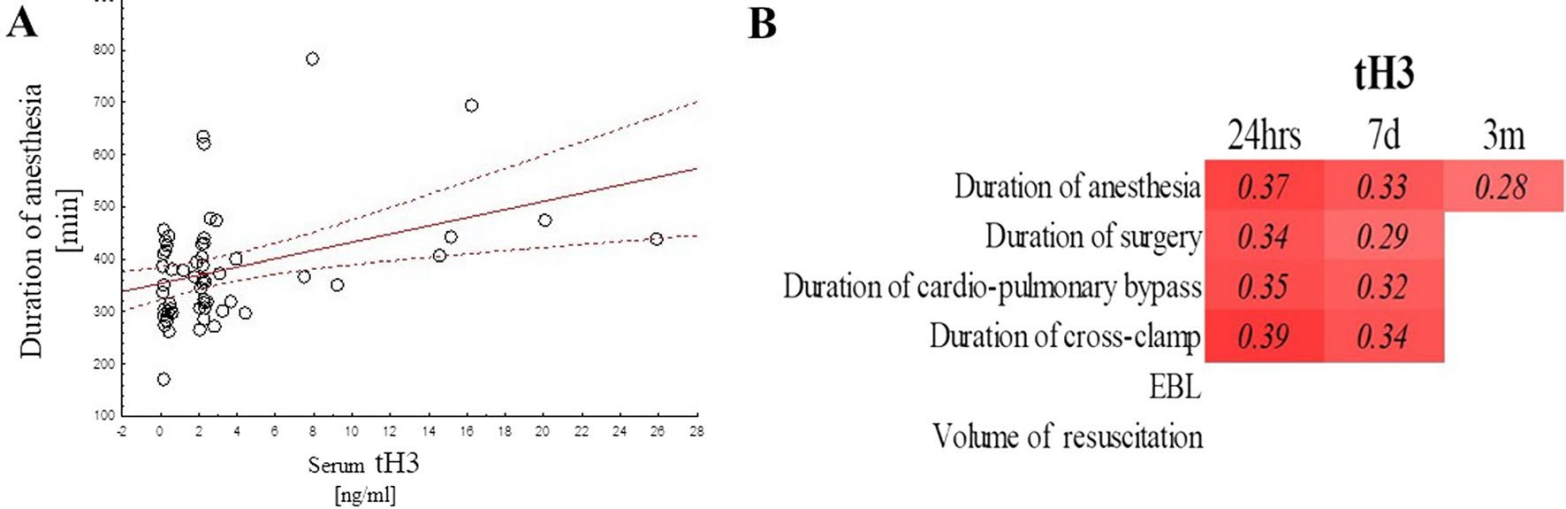
tH3 serum level and peri-operative events. There was significant correlation between tH3 and duration of anesthesia **A** as well as other measures of the burden of surgical intervention **B** with only significant (*p* < 0.05) presented on figure B

The type of surgery (CABG, aortic valve surgery, mitral valve surgery, arch or aneurysm surgery, or other surgery) did not affect serum tH3, H3K4me3, or H3K27ac in the post-operative period (data not shown). When examining correlations between anesthesia, surgery, and peri-operative parameters with serum levels of tH3 and its modifications, the serum levels of tH3_24hr_ and tH3_7d_ correlated highly with the duration of anesthesia (Fig. 2A and B), duration of surgery (Fig. 2B) time on bypass but not the amount of estimated blood loss, crystalloid given, or blood products (Fig. 2B). Serum tH3_3m_ only correlated with the duration of anesthesia and surgery (Fig. 2B).

Serum H3K27ac at 24 h correlated with the volume of crystalloids (*r*^2^ = 0.46; *p* < 0.05) and packed red blood cells (PRBC) (*r*^2^ = 0.37; *p* < 0.05) used for resuscitation during anesthesia. H3K27ac at seven days correlated with the volume of PRBC (*r*^2^ = 0.37; *p* < 0.05). The serum level of H3K4me3 failed to demonstrate any difference between surgical parameters and their serum level at any time;

Peri-operative intake of acetaminophen significantly decreased serum levels of H3K4me3 at 24 h (H3K4me3_acetom-_ = 1.26 [0.71;3.21] vs. H3K4me3_acetom+_ = 0.54 [0.07;1.01]; *W*[50] = 2.26; *p* = 0.021), although the correlation between the dose and the serum level of H3K4me3 was below statistical significance. Neither acetylsalicylic acid, ketorolac, or steroid intake affected tH3, H3K4me3, or H3K27ac levels at any studied time point (data not shown).

### Evolution of general inflammatory markers (IL-6, CRP) in the aftermath of cardiac surgery and their relationship to serum histone

**Fig. 3:**
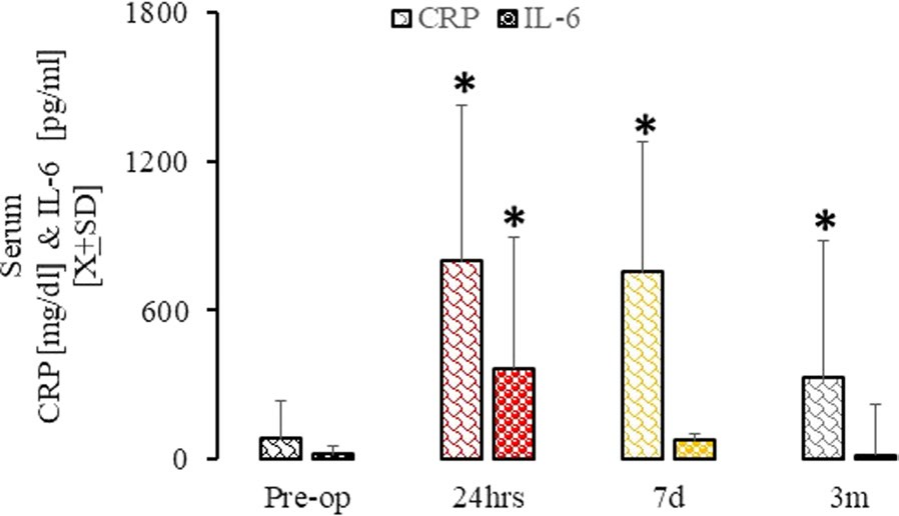
General inflammatory markers pre- and post-cardiac surgery. Serum CRP (dark scales) and IL-6 (dark bubbles) levels before surgery (t_Pre-op_), and 24 h (t_24h_), 7 days (t_7d_), and 3 months (t_3m_) after surgery. *p < 0.05 as compared to t_pre-op_

General inflammation was measured with serum levels of IL-6 and CRP. Compared to pre-surgical levels, serum IL-6 levels were significantly higher 24 h after surgery but normalized at seven days and three months (Fig. 3). In contrast, CRP levels were significantly elevated at all three time points, but levels at three months were significantly less than those at 24 h and seven days after surgery (Fig. 3). Neither CRP nor IL-6 serum levels correlated significantly with duration of anesthesia, duration of surgery, or time on bypass or cross-clamp (data not shown).

HSP-60 was elevated 24 h after surgery, but at 7 days and 3 months post-surgery, the levels drop to below pre-surgical values (HSP60_Pre-op_ = 3.4 ± 8.52; HSP60_24hrs_ = 6.4 ± 16.1; HSP60_7d_ = 2.1 ± 5.01; HSP60_3m_ = 2.0 ± 5.35).

**Fig. 4:**
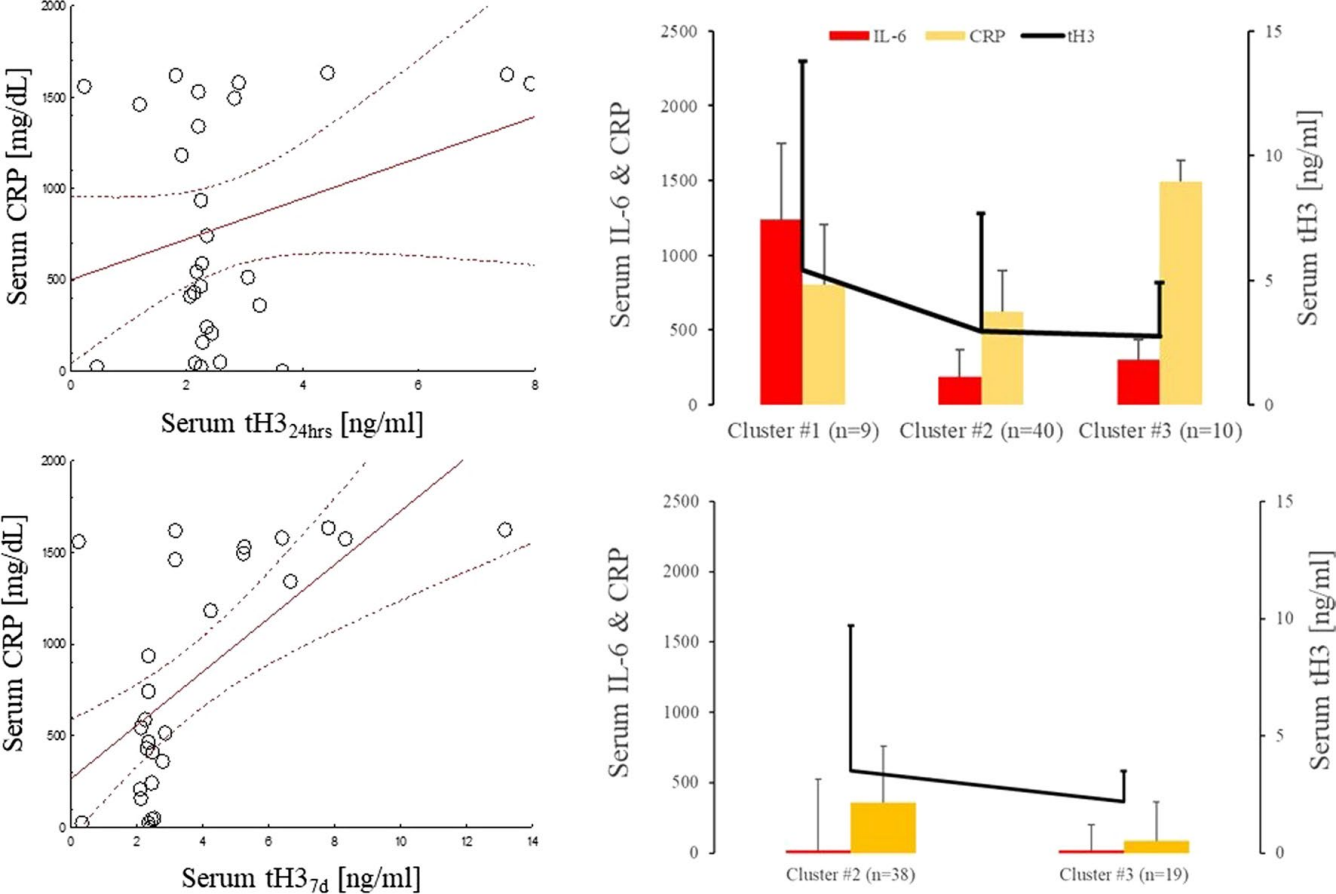
General inflammatory markers pre- and post-cardiac surgery. A Correlation between tH324h and CRP at 24 h (**A**) and 7 days (**B**). Cluster analysis of patients grouped the individuals at 24 h into clusters #1, 2, and 3 (**C**). At long term follow-up only two clusters were seen, one with persistent inflammation containing patients (**D**). Levels of IL-6 (red), CRP (yellow), and tH3 (black line) are indicated

When examining correlations between general inflammatory markers and serum levels of tH3 and its modifications, no significant correlations were observed between levels of serum IL-6 and tH3, H3K4me3, or H3K27ac. There were significant correlations between CRP_24hr_ and tH3_24hr_ (*r*^2^ = 0.43; *p* < 0.05) (Fig. 4A), tH3_7d_ (*r*^2^ = 0.71; *p* < 0.05)(Fig. 4B), H3K4me3_7d_ (*r*^2^ = 0.53; *p* < 0.05), and H3K27ac_7d_ (*r*^2^ = 0.49 *p* < 0.05). Cluster analysis revealed three distinct groups of patients 24 h post-surgery (Fig. 4C). Cluster #1 (*n* = 9) had the highest serum levels of CRP, IL-6, and tH3. Cluster #2 (*n* = 40) and Cluster #3 (*n* = 10) both had lower levels of tH3 than Cluster #1, but their serum CRP levels were different, with Cluster #3 having higher levels than Cluster #2 (Fig. 4C). At three months, cluster analysis revealed only two distinctive groups (Fig. 4D). Cluster #1 (*n* = 38) contained patients with higher levels of tH3 and CRP than Cluster #2 patients (n = 19).

### Histone release patterns after whole blood stimulation.

**Fig. 5:**
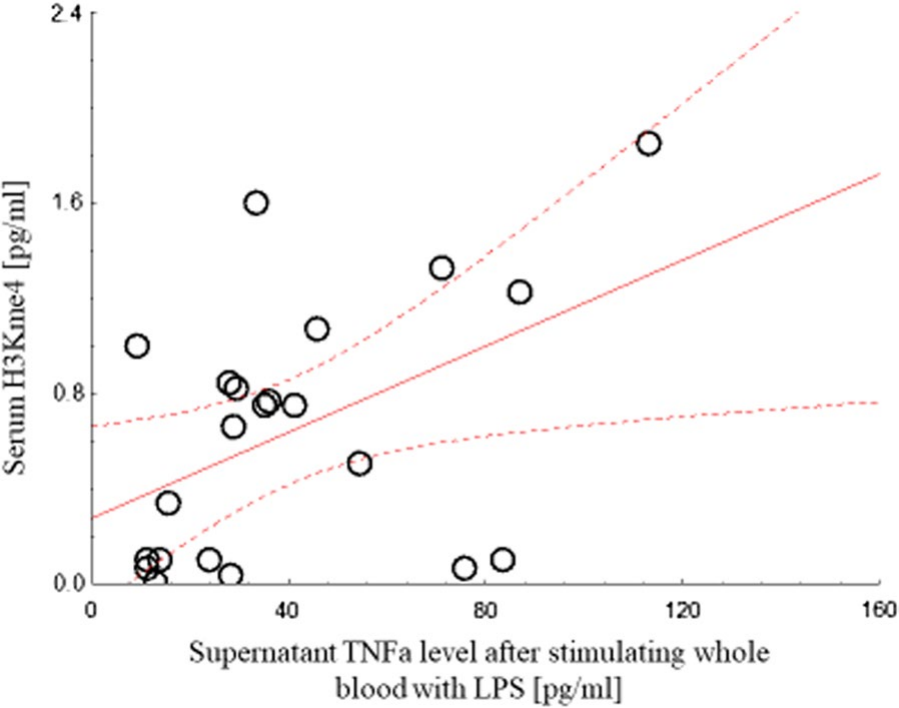
Correlation between leukocyte activation and serum H3K27ac. A significant correlation is seen between TNFα production in response to LPS by whole blood and serum H3K27ac levels at 24 h post-surgery

In unstimulated blood, the production of TNFα at 24 h significantly correlated with H3K4me3_24hr_ (*r*^2^ = 0.96; *p* = 0.000), but not with tH3_24h_ or H3K27ac_24h_. At 24 h post-surgery, the production of TNFα in LPS-stimulated blood obtained at the same time significantly correlated with serum levels of tH3_24h_ (*r*^2^ = 0.67; *p* = 0.001) (Fig. 5). The production of IL-6 by LPS-stimulated whole blood did not correlate with tH3, H3Kme4, or H3K27ac serum levels (data not shown). The serum levels of CRP and HSP-60 did not correlate with the production of TNFα or IL-6 by LPS-stimulated whole blood at any time.

### Correlation of serum histone levels with post-operative clinical characteristics

The length of stay in hospital correlated with tH3_24hrs_ (*r*^2^ = 0.29; *p* = 0.027) and the length of stay in the ICU correlated with H3K4me3_24hrs_ (*r*^2^ = 0.34; *p* = 0.013). There were no significant correlations between APACHE scores at admission to the ICU or 24 h or 48 h and any of the histones (data not shown).

**Fig. 6:**
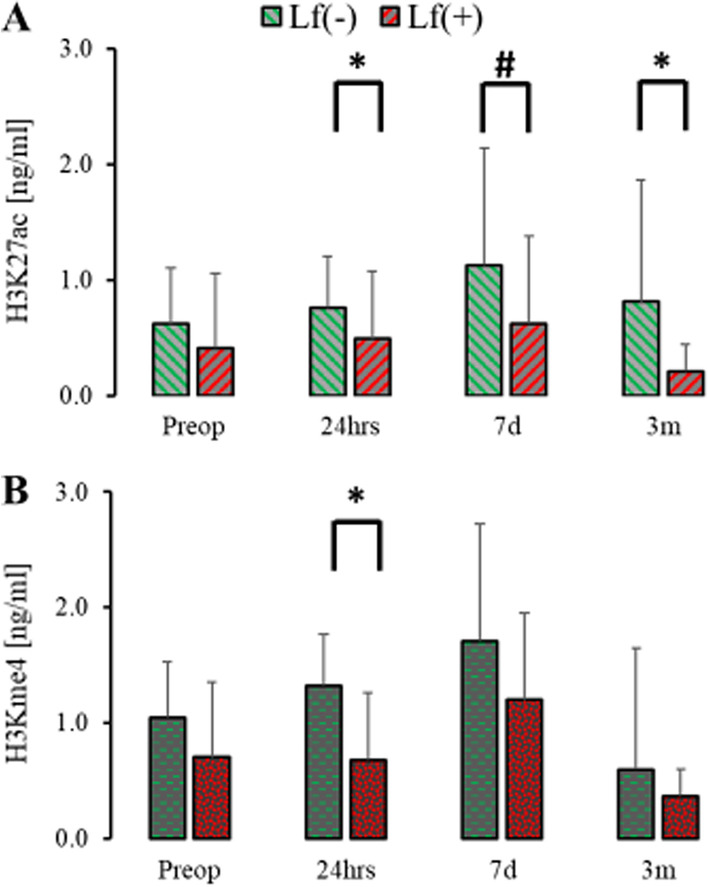
Histone levels in patients with and without liver failure. Levels of H3K27ac were elevated at 24 hours, 7 days amd 3 months post-surgery in patients with liver failure [Lf( +)] as compared to those and without liver failure [Lf(-)]. In contrast, H3Kme4 was significantly different only at 24 h. *p < 0.05 for two-sided hypothesize, #*p* < 0.05 for one-sided hypothesize

Patients with liver failure defined along MODS criteria at 24 h showed significantly diminished serum levels of H3K27ac at 24 h, seven days, and three months as compared to those without liver failure (Fig. 6A) [52]. Serum H3Kme3 was depressed only at 24 h if the lever failure was apparent in the peri-operative period (Fig. 6B). Serum tH3 did not differ between patients with or without liver failure. The emergence of the central nervous system(CNS_F_), respiratory failure (R_F_) or acute renal failure (AKI_F_) as defined by MODS definitions did not affect the serum level of measured histones at any point [52]. Mortality and the rate of other complications (PE, DVT, CVA) were too low to conduct rational statistical analysis.

## Discussion

We demonstrated for the first time the presence of circulating histone three months after elective heart surgery among adult individuals. Previous data were limited to three days of follow-up and focused on children undergoing surgery for congenital heart disease [23].

The initial presence of tH3 signifies the necrosis and apoptosis secondary to iatrogenic surgical insult [3, 8, 21, 22]. Similar histone leakage was seen in other severe critical care illness cardiomyopathy [9]. Concomitant increase of serum HSP-60, a DAMP being released from cells upon destruction, supports this idea, and it was seen in cardiac surgery injury before [21, 22]. More pronounced elevation in serum histone in younger patients suggests that DAMP release is proportional to the tissue's total volume. The lack of correlation with troponin suggests that the release of circulating histones is predominantly secondary to iatrogenic surgical injury instead of acute myocardial ischemia [12, 53]. Correlations between time for anesthesia, surgery, bypass, and cross-clamp with serum tH3 during hospitalization were significant, suggesting that duration of surgical exposure is the critical factor for histone release [9, 12, 22, 23]. The lack of significant differences among various types of surgeries suggests that the degree of tissue damage is the critical factor, not the anatomical organ. Also, different etiologies leading to surgery seemed not to be that impactful in acute post-cardiac surgery histone dynamics despite playing a significant role in etiology leading to surgery [26, 46]. This is not surprising considering that circulating histones in the peri-operative period are markers of tissue destruction instead of being markers for functional or homeostatic status [18, 25, 33, 46]. However, the presence of persistent serum histones at three months is puzzling. The concomitant persistence of CRP suggests that ongoing smoldering inflammation is the potential source of tH3 in serum, as a rapid decrease in IL-6 suggests that acute inflammation is mainly resolved past seven days [16, 39]. Quick resolution of the initial serum elevation of Hsp-60 would indicate that the necrotic process secondary to ischemic/reperfusion injury mainly occurs within seven days and is unlikely to contribute to tH3 serum leakage months after surgery [21, 22]. Consequently, we suggest that serum tH3 at three months serves as a danger or remodeling signal [5, 7–9, 55, 56]. The source of tH3 is unclear, but heart remodeling and increased leukocyte turnover are other potential sources of increased histones in serum [3, 55, 57]. This finding challenges the common assumption that the post-surgical recovery period is complete within the traditionally established period of 28 days [1, 2, 15, 16, 49].

We demonstrated that both H3 and CRP have prolonged presences in serum after cardiac surgery. However, the clinical importance of this finding is unclear. IL-6, CRP, and tH3 coalesce along the initial cluster to demonstrate unique patterns early after surgery and late in convalescence. Considering the complex interplay between these factors, the deployment of clustering techniques revealed higher order data that otherwise would not be apparent. The complex interplay between these factors stems from multiple roles of CRP, IL-6, and tH3, which are very context and level dependent [5–7, 20, 38, 39, 41–44, 58, 59]. CRP serves as a protective moderator of toxic histone presence but only if lethal doses of histones were used in the study in settings quite different from our study design [38]. We also did not study other protective mechanisms against histone toxicity, but if these mechanisms are depleted, the adverse effect of circulating tH3 may be exacerbated [35, 36, 60, 61]. Extracellular circulating histones trigger coagulation cascade and increase endothelial permeability, all of which were present in a few of our or were not studied [5, 7, 8, 10]. However, the clinical consequences of histone toxicity are numerous (pancreatitis, lung failure, pulmonary embolism, deep venous thrombosis, and stroke) [5–10, 13, 36, 57]. Our study was not powered to demonstrate the clinical outcomes regarding circulating histone. Since histone and CRP have a complex relationship in the acute peri-operative period, the clinical importance of the interplay between histone and CRP needs to be established [38, 62]. This is particularly important considering that our cluster analysis showed that IL-6, CRP, and tH3 coalesce in distinctive subpopulations over time. Therefore, future analysis should include the composite picture of tH3, CRP, and other factors to determine the optimal milieu for recovery or the most detrimental composition of the factors mentioned above in the acute period. The importance of simultaneous and prolonged elevation of tH3 and CRP outside acute inflammation is likely to be different compared to acute one. CRP is produced during the inflammatory process, serving as an immunomodulator and marker of smoldering inflammation [6, 39, 43]. Considering both histones and CRP in vascular inflammation and arteriosclerosis, their prolonged presence may suggest accelerated atherosclerosis in the wake of cardiac surgery [25, 26, 40, 42–44, 46, 63]. Consequently, addressing delayed post-surgical inflammation and persistence of DAMP may suggest a more aggressive approach to limit post-surgical sequela [39, 63].

We explored whether histone modification may be linked with the emergence of the immunological status in patients undergoing heart surgery [7, 47]. Prior studies had focused on modifying histone three from MO in patients with coronary artery disease, but the sample was small [20]. We observed that H3K4me3 and H3K27ac had different time dynamics after heart surgery. H3K4me3 initially increased at 24 h but recovered to baseline levels at three months, while H3K27ac increased at seven days and diminished at three months. These histone modifications are linked to the differential activation of the immune system [4, 23, 47]. H3K4me3 is linked to multiple early immune activators [33]. H3K27ac has several immunomodulatory-related activities [59]. The changes in the composition of circulating histone may reflect immune system performance. Interestingly, the increased response to LPS stimulation by circulating leukocytes correlated with serum H3K4me3. This suggests that an increased inflammatory response from leukocytes is reflected by elevated H3K4me3, but a more in-depth study is needed to confirm a causal relationship [47, 64, 65]. The significance of this finding is that the abnormal release of TNFα in response to LPS may be linked to a post-operative “second hit” and delayed organ failure [1, 14–16, 27].

Several medications exhibit frequently underappreciated epigenetic activities [29, 30]. We did not observe an effect of opioids on pre- or post-surgical changes in histone modification. Other compounds, such as cocaine and cannabinoids, were not studied since patients that have taken these compounds would be disqualified from surgery [66]. Several other medications, such as antidepressants, hydralazine, anti-seizure, and others, were also not surveyed [30]. However, we found that acetaminophen intake was linked to the depressed serum of H3K4me3 changes during the acute peri-operative period. Some data suggest an effect of acetaminophen on DNA methylation [31, 67, 68]. DNA methylation affects histone modification. Alternatively, acetaminophen could suppress inflammation via inhibitory cyclooxygenase or its active metabolites acting via transient receptor potential vanilloid 1 (TRPV1) and cannabinoid 1 (CB1) receptors [69, 70]. This immunosuppression is then reflected in H3K4me3, which is linked to immune activation [33, 69]. This finding further underscores the importance of frequent acetaminophen use for postoperative pain management, often in conjunction with other epigenetic modifiers like caffeine and opioids [30, 69]. Finally, the decrease in H3K4me3 is not related to acetaminophen-mediated liver toxicity since that one resembles sterile inflammation [13, 69, 71]. Our study implies that changes in histone modification mediated by a singular dose of the peri-operative medication may modulate the emergence of late complications [72, 73].

In contrast to prior research, we did not demonstrate a correlation between elevated serum levels of histones and several features of clinical demise [6–8, 10, 11, 13, 57, 73]. Several factors may account for that, but patient selection and management may be the most important. Cardiac surgery patients studied here undergo protocolized procedures to be extubated within 24 h, with over 85% achieving this goal. Consequently, traits of typical acute respiratory distress syndrome are rare. Most of the data describing the role of histones in respiratory failure were done in animals or patients developing acute respiratory distress syndrome (ARDS) secondary to sepsis [6–8, 12]. We did not have a single episode of sepsis in our studied population. Our data may suggest that either histone release in cardiac surgery is insufficient to trigger ARDS, protective mechanisms are sufficient to moderate the toxic effect of histone release, a mechanism of lung injury secondary to cardiac surgery requires multiple DAMP releases or peri-operative congestive heart failure of the left ventricle results in different histone release dynamics [8, 13, 34, 36, 38, 60, 61]. The latter is unlikely as the peri-operative levels of NT-BNP, a marker of congestive heart failure did not correlate with serum histone levels, suggesting that chronic and acute cardiac dysfunction may have a different effect on histone release [54]. Some researchers utilized the composite score of all adverse events, but we use the framework on the MODS, allowing for precise delineation of the failure [23].

We found that the emergence of liver failure was related to a decrease in the level of total histone 3, while prior studies demonstrated that liver failure was related to increased serum histone, but these studies examine liver injury as the primary source of the insult [5]. In our study, liver failure was related to liver congestion and right ventricular failure, and the resultant liver injury gave rise to changes in methylation instead of the total amount of tH3, suggesting that the inflammatory process is reflected in this change instead of histone toxicity being the driver of tissue injury [5, 13]. A preserved level of CRP suggests that synthetic liver function was not impaired. Finally, histone-mediated organ failure in prior studies has been assessed in prolonged and severe diseases like sepsis, drug-induced liver failure, and pancreatitis [5, 7–9]. In contrast, cardiac surgery creates a moderate but prolonged insult in most patients, providing a very different exposure to tH3. We demonstrated a rapid decrease in histone levels, lower than those observed in acute infectious diseases [9, 11, 19, 53].

The interesting clinical finding of this study is the perseverance of circulating histones well into recovery after surgery, which may contribute to the emergence or acceleration of atherosclerosis [39, 44]. In addition, extracellular histones may act as DAMP, promoting endothelial dysfunction and vascular inflammation. Concomitant elevation in CRP may provide another synergistic stimulus for the progression of atherosclerosis [23, 44, 63, 74, 75]. Designing a targeted intervention based on the extracellular histone profile using blood as the biospecimen source has the advantage of sample availability. Atherosclerosis is modulated by several perivascular structures (endothelium, myocardium, and epicardial fat) during acute peri-operative stress and post-operative convalescence. However, utilizing blood may help to identify the specific epigenetic patterns guiding the effective therapy [57].

This study's longitudinal design accounting for pre-operative baseline measurements is a strength. This longitudinal analysis allowed for comparing the changes in histone levels and modifications between baseline and follow-up measurements for individual patients as the epigenome is highly variable inter-individually [32, 33, 46, 76]. This study included a relatively large adult patient population with highly homogenous peri-operative care. Our study controlled for age and specific drug intake, assessing their influence on serum histone levels [25, 29, 30]. Several comorbidities were accounted for, including heart dysfunction and end-stage renal disease [77]. We did not include patients with a neoplasm or undergoing chemotherapy.

Future studies must incorporate a significantly larger study sample to correlate circulating histone levels with organ failure. In addition, diet and tobacco exposure would need to be incorporated into analyses to control for epigenetic mediators. Finally, focusing on a subset of cardiac surgeries will reduce the heterogeneity of the insult, as we saw the difference related to age which is likely related to surgical intervention.

## Conclusions

We demonstrated a prolonged elevation of serum histone three levels after cardiac surgery in an adult population. Furthermore, histone three modifications had a discrete time evolution and correlated with the activation of the immune system at 24 h. The dominant factors in histone elevation were related to the duration of surgery and not etiology leading to the procedure. The relationship between changes in H3Kme4 and monocyte-altered responsiveness to bacterial pathogens necessitates further study to demonstrate whether this increases susceptibility to organ failure.

## Figures and Tables

**Fig. 7:**
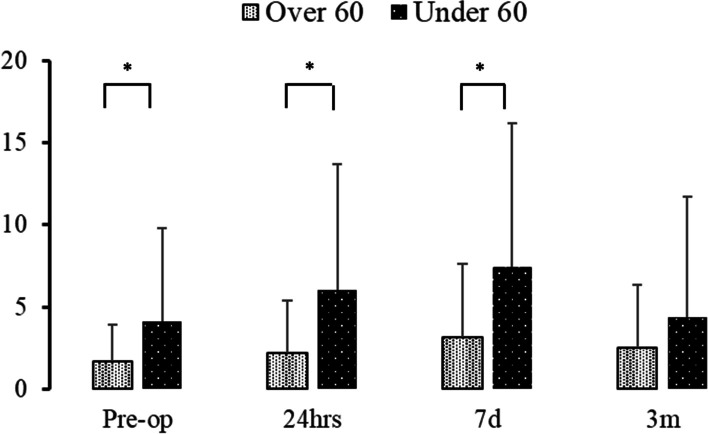
Changes in tH3 level between older (over 60 years old) *and* younger. Younger subjects demonstrated elevated levels of circulating histone 3 before the surgery, 24 h and 7 days but not three months. *denotes *p* < 0.05 when comparing both age populations

## Data Availability

The datasets used and/or analyzed during the current study are available from the corresponding authors on reasonable request after IRB’s approval.

## Appendix

See Fig. 7
